# Multicentre Retrospective Cohort Study on Current Practices in Treatment of Patients Presenting with Non-A Non-B Aortic Dissection and Factors Predicting the Need for Intervention and Mortality

**DOI:** 10.3390/jcm15010211

**Published:** 2025-12-27

**Authors:** Ottavia Borghese, Gabriel Lopez-Pena, Athanasios Saratzis, Tryfon Vainas, Alice Lopes, Blandine Maurel, Tara Mastracci

**Affiliations:** 1 Department of Cardiothoracic Surgery, St. Bartholomew’s Hospital, London EC1A 7BS, UK; 2 Unit of Vascular Surgery, Fondazione Policlinico Universitario A. Gemelli IRCCS, 00168 Rome, Italy; 3 Leicester Vascular Institute, University Hospitals of Leicester NHS Trust, Leicester LE1 5WW, UK; 4 Department of Cardiovascular Sciences, NIHR Leicester Biomedical Research Centre, University of Leicester, Leicester LE1 7RH, UK; 5 Heart and Vessels Division, Hospital de Santa Maria (ULS), 1649-035 Lisbon, Portugal; 6 Service de Chirurgie Cardio-Vasculaire, L’institut du Thorax, CHU Nantes, Université de Nantes, 44307 Nantes, France; 7 Department of Surgery and Interventional Sciences, University College London, London WC1E 6BT, UK

**Keywords:** acute aortic syndromes, Non-A Non-B aortic dissection, frozen elephant trunk technique, total arch replacement, TEVAR, outcomes

## Abstract

**Objectives:** Non-A Non-B (NANB) aortic dissections (ADs) are uncommon. Because of their rarity, their therapeutic pathway is not yet standardized, and anatomic or goal-directed treatments are not reported in current practices. We reviewed the treatment strategies of NANB AD across Europe, aiming to identify factors associated with increased mortality and the need for intervention, outlining optimal management pathways for future care. **Methods:** This multicentre cohort study was carried out in four European aortic centres, retrospectively including patients affected by NANB AD over the last 10 years. Patients’ anatomical clinical and treatment data were collected with the aim of investigating the factors associated with their need for intervention and increased mortality, comparing the characteristics of those requiring surgery with those who responded to medical treatment alone. **Results:** Thirty-eight NANB patients (26, 68.4% men; mean age 60.6 ± 12.87) were included. The primary entry tear was identified in Ishimaru zone 1 or 2 in most cases (24, 63.2%) and the dissection extended distally to the ilio-femoral arteries in half of the patients (21, 55.3%). Surgical repair was indicated in 21 (55.3%) cases within 90 days of acute onset for end-organ ischemia, impending aortic rupture, or retrograde extension of the dissection (including 11 emergent/urgent operations), with most patients requiring surgery within 15 days of acute onset (17, 44.7%). The mean aortic diameter among patients requiring surgery was significantly higher in both zone 1 (7 37 IQR 3 versus 34 IQR 7, *p* = 0.043) and 2 (36 IQR 6 versus 32.5 IQR 7, *p* = 0.044) when compared with patients who underwent medical treatment alone. An increased in-hospital mortality rate was noted among patients with indication for surgery after medical treatment (0% versus 30.8%, *p* = 0.023). **Conclusions:** This cohort provides an additional description of clinical aspects and current practices in the treatment of NANB in Europe. Most patients of this series had an indication for surgery within two weeks of acute onset, demonstrating a frequently complicated course; moreover, this raises questions surrounding the most appropriate timing for interventional management. Although a diameter threshold was not identified, the baseline enlarged aortic diameter in zones 1 and 2 seemed to be associated with a need for early intervention. Further study is needed to fully refine the indications for treatment in NANB patients; this will support the study of the independent risk factors for increased mortality risk and complications among this group, and will allow the identification of subgroups of patients that may benefit from more aggressive treatment from acute onset.

## 1. Introduction

Non-A and Non-B (NANB) represent a small percentage of aortic dissections (ADs) (with a reported incidence of about 3–11%). The term NANB was firstly introduced in 1994 by von Segesser and colleagues, identifying AD cases with a primary tear in the arch [1]; however, proper categorization was lacking till the more recent introduction of the TEM (Type, Entry, Malperfusion) approach and SVS/STS classification. This meant that NANB cases were frequently unrecognised and classified as type B [2,3,4]. 

Currently, “NANB” describes a heterogeneous group of AD cases that may deeply differ in terms of clinical and anatomical presentation, but which can all be characterized by the involvement of the aortic arch, which may be the site of the primary entry tear or may be retrogradely affected [5,6]. 

Clinical presentation may vary greatly; it has been reported that NANB cases more frequently display a complicated course to the extent that most patients will be operated upon within two weeks of presentation—this is due to the proximal progression of the dissection or as an attempt to treat complications [7]. 

The most recent meta-analysis on this subject demonstrated that the 30-day mortality rate of medically managed patients is three-fold higher than that of those interventionally treated [7]; however, because of the rarity of these cases, a therapeutic pathway has not yet been standardized, and anatomic or goal-directed treatments have not been reported in current practices. Both the European and American vascular guidelines strongly push toward individualised treatments in high-volume specialised aortic centres, where staff are skilled in multidisciplinary decision making and have expertise in both open and endovascular management [4,6]. 

Currently, the management strategy seems to be influenced by the subspecialty of the treating physician, varying from extensive open surgical replacement to all endovascular approaches, regardless of the site of the primary tear [8,9,10,11]. 

Nevertheless, because the reported data are heterogenous, it is difficult to definitively compare the outcomes achieved with open versus endovascular strategies. Regardless, the currently available data demonstrate an increased 30-day mortality rate among patients treated medically versus those who undergo intervention (14% versus 3.6%) [7]. 

In this context, we reviewed the treatment practices for NANB acute AD across four high-volume aortic centres in Europe with the aim of investigating the factors associated with the need for intervention and increased mortality; we hope to contribute to a broader understanding of the optimal management of this uncommon disease in future care.

## 2. Materials and Methods

### 2.1. Study Design

This was a retrospective, multicentre, international, observational study carried out in four high-volume aortic centres in Europe between 1 October 2022 and 30 April 2023; all patients hospitalized for an NANB AD over the previous 10 years were included.

The included centres were the St. Bartholomew’s Hospital (London, UK), the Glenfield Hospital (Leicester, UK), Santa Maria Hospital (Lisbon, Portugal), and the Nord Laennec Hospital (Nantes, France).

Each local authority approved the study at the single participating centre as an audit for service improvement. All data were collected in an anonymized database; due to the retrospective observational nature of this study, formal ethics approval was waived. All patients gave informed consent to the operation and to the anonymized collection and publication of their clinical data and imaging.

NANB was defined as an AD with a primary entry tear in Ishimaru zones 1 and 2 or more distally but with retrograde extension into the arch [1,2]. Retrograde intramural hematoma was considered as an extension of the dissection as well.

Patients were enrolled according to the following inclusion criteria: (a) age > 18 years; (b) acute (<15 days) onset of type NANB AD; (c) diagnosis made by 1–3 mm cuts in contrasted computed tomography angiography (CTA).

All other acute aortic syndromes (including type A/B AD, penetrating aortic ulceration, intramural hematomas, aneurysm, or aortic trauma and traumatic dissection) were excluded.

Standard protocol for CTAs at acute onset included a pre-contrast scan before the biphasic injection of iodinated contrast material, coverage from supra-aortic vessels to the groin using a 64-detector CT scanner, and section thickness between 1 mm and 2.5 mm.

**Figure 1:**
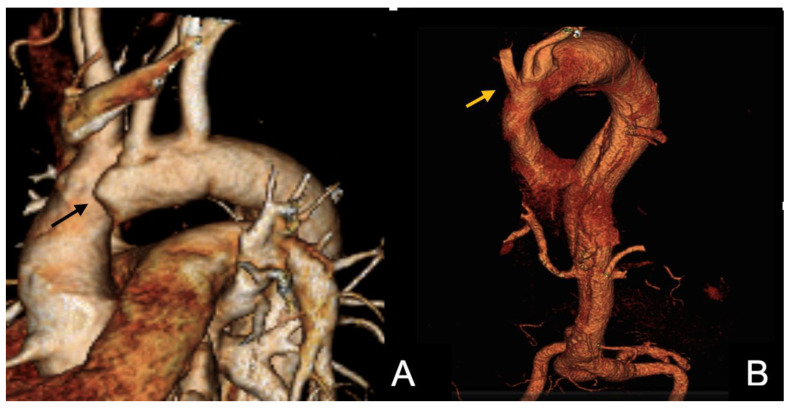
volume rendering of the baseline computed tomography angiography (CTA) of two patients included in this series. The arrows indicate the primary entry tear in Ishimaru zone 0 (**A**) and zone 1 (**B**).

The CTAs at admission were reviewed to retrieve data on the location of the proximal tear, the extent of the dissection, the involvement of collateral branches, and the presence of anatomical abnormalities (bicuspid aortic valve, Kommerel diverticulum, bovine arch, anomalous origin of supra-aortic vessels) (Figure 1).

The baseline aortic diameters in Ishimaru [12] zones 1–7 were also calculated.

Serial CT examinations were performed in all survivors of acute onset (after 1–3 months, 6 months, and yearly thereafter).

The protocol for CTA analysis was standardized across the four participating Institutions according to the current reporting standard on type B dissections [2] and imaging analysis was performed using the Aquarius workstation (TeraRecon Inc., Foster City, CA, USA) for postprocessing.

The anatomy of the AD and end-organ perfusion were clinically and radiologically assessed in all patients on admission. Patients initially presenting with non-complicated dissection (hypertension or pain only) were admitted to hospital for blood pressure optimization and analgesia and underwent interval CTAs for surveillance of dissection anatomy and complications; these intervals were chosen according to timing that varied between 24 and 48 h after acute onset across the different institutions.

This approach is indicated as the best medical treatment (BMT). Conversely, patients presenting with end-organ ischemia, signs of impending aortic rupture at the CTA (crescent sign, focal wall discontinuity of circumferential calcifications, and aortic bulges or blebs), or retrograde extension of the dissection into the ascending aorta or root were interventionally treated on emergency basis.

End-organ ischaemia was defined as the combination of clinical (pulseless/cold extremities, severe abdominal pain), laboratory (elevated serum lactate, increased creatinine, and liver function test levels), and imaging (collapsed true aortic lumen, dissected visceral arteries with significantly narrowed true lumen by a thrombosed false lumen (FL)) evidence [2]. 

Patients suffering with ongoing clinical symptoms, raising lactate or creatinine level, with continued true lumen compression/lack of opacification of the mesenteric/renal vessels or lower limb were scheduled for surgery. The decision on the type of interventional strategy (endovascular, hybrid or open) was made after multidisciplinary discussion at the each treating centre.

### 2.2. Endpoints

Our primary outcome was to investigate the factors associated with need for intervention and increased mortality among patients with NANB AD.

Therefore, we reviewed the practice in treatment of NANB acute AD across four high-volume aortic centres in Europe, comparing the clinical and anatomical characteristics of patients who had indication for surgery or who received medical treatment only.

The following clinical data were retrieved from the electronic records at the participating institutions:

- Demographics (age, gender, ethnicity); cardiovascular risk factors (diabetes, hypertension, obesity, smoking status, recreational drug use).
- Coexisting morbidities (renal, cardiac, cerebrovascular, pulmonary, peripheral arteries diseases and congenital aortic syndromes).
- Clinical presentation at the acute onset.
- Selected treatment strategies and complications encountered.
- In-hospital mortality (death during the first hospitalization).

The mid-term mortality and the need and indication for intervention during follow-up were assessed during outpatient visits, retrieved from the electronic health records at each institution or by telephone contact.

### 2.3. Statistical Analysis

The categorial variables are reported in percentages and absolute values. Continuous variables are expressed in mean with standard deviation (SD) or in the median and IQR (interquartile range) of the values obtained.

The Chi squared test or Fisher’s exact test were used for categorical variables; the unpaired t-test was used for comparing the means of the continuous variables, and the Mann–Whitney U test was used for comparing the medians of the continuous variables.

Univariable analysis was performed to identify the independent predictors of need for intervention and 30-day and mid-term mortality. The variables identified in prior clinical evidence and with *p*-values < 0.25 in the univariable analysis (by setting a higher significance level to enable more variables to show their significance) and were included in a multivariable analysis performed with the ‘enter’ method. No stepwise procedures were used.

Kaplan–Meier survival curves estimated survival during follow-up. *p*-values < 0.05 were considered statistically significant for all analyses.

Statistical analysis was performed with SPSS v.25 statistics software (SPSS Inc., Chicago, IL, USA).

## 3. Results

### 3.1. Baseline Data and Management Strategies

A cohort of 38 consecutive patients (26 male, mean age 60.6 ± 12.9), including either dissections beginning at the aortic arch (24, 63.2%) or descending aorta dissections with retrograde involvement of the arch (14, 36.8%) was analysed.

Main comorbidities and cardiovascular risk factors are reported in Table 1. One patient (2.6%) had a tissue connective disorder (Marfan syndrome) and most (35, 92.1%) had a history of hypertension under medical treatment.

Upon admission, patients displayed a combination of one or more symptoms, including hypertension (24, 63.2%), chest/back or abdominal pain (34, 89.5%), neurological symptoms (3, 7.9%), shortness of breath (1, 2.6%), or syncope (2, 5.3%).

Signs of end-organ ischemia (renal/lower limbs ischemia) were detected in only four (10.5%) cases at acute onset.

One patient presented with a bovine arch, but no other anatomical abnormalities were reported. Further anatomical details are reported in Table 2.

The primary entry tear was identified in Ishimaru zone 1 in 7 cases (18.4%), in zone 2 in 17 cases (44.7%), and in zones 3 or 4 in 14 cases (36.8%).

The aortic dissection extended proximally to zone 1 in 13 (34.2%) cases or to zone 2 in 25 (65.9%) cases; distally, the disease reached zone 2 in 2 (5.3%) cases, zone 3 in 1 (2.6%) case, the visceral aorta in 5 (13.2%) cases, the infrarenal aorta in 9 (26.7%), and the iliofemoral arteries in the remaining 21 (55.3%) cases.

A mean of 1.5 (range 0–5) branches were noted that had split origin or which totally originated from the false lumen.

All patients received BMT upon admission and were scheduled for surgery according to the criteria reported above. Overall, seven patients (18.4%) required an emergent operation (identified following as the interventional group) but most of them (31, 81.6%) responded to medical treatment only (described herein as the BMT group). A mean of 2.3 (range 1–5) antihypertensive drugs were used to achieve systolic blood pressure < 110 mmHg systolic (mean arterial pressure—85 mmHg).

However, within 90 days from the acute onset, overall, 21 (55.3%) cases finally had indication for surgery.

Reasons for intervention were one or more of the following: hemodynamic instability, persistent pain in the setting of low blood pressure, risks of impending aortic rupture, secondary retrograde extension to the ascending aorta/root; end-organ ischemia.

In detail, indications for surgery in 21 cases were given as follows:

- Urgent operations were performed in seven (18.4%) cases as reported above;
- Ten (26.3%) patients required surgery within 15 days (mean, 13 days; range, 0–30 days) for recurrent symptoms or uncontrolled hypertension with or without imaging evidence of proximal extension of the AD (7) or end-organ ischemia (1).
- Two (5.3%) patients were operated upon within two months (27 and 43 days after the initial diagnosis, respectively) for recurrent pain and proximal extension at the CTA.
- Finally, two patients died from aortic rupture and cardiac tamponade one and two days after the acute onset, respectively, while waiting for surgery. In those cases, dissection originated from zones 3 and 2, respectively, and extended to the iliofemoral vessels; aortic diameter at the entry tear site was 35 mm in both cases, with a dilated non-dissected ascending aorta (maximal diameter 46 mm) being reported in one case (Figure 1).

Interventional strategies were performed with the aim of covering the entry and the additional distal tears when identifiable. These included traditional open surgery with conventional arch replacement (AR) (two urgent cases, 5.3%), a frozen elephant trunk (FET) procedure (12, 31.6%; of these, 3 were urgent cases), hybrid strategies with zone 1 debranching and TEVAR (4, 10.2%; of these, 1 was an urgent case), or ascending repair and Ascyrus Medical Dissection Stent (AMDS, Cryolife/Jotec, Hechingen, Germany) implantation (1, 2.6%, which was an urgent case).

Six additional procedures were required at the time of the first operation to treat lower limb ischemia (one femoro–femoral and two axillo–bifemoral bypass), aortic valve insufficiency (two aortic valve replacements), coronary artery stenosis (one bypass), and kidney malperfusion (one stenting procedure) (Table 3).

### 3.2. Clinical Results and Factors Associated with Mortality and Need of Intervention

The in-hospital mortality rate was 13.2% (five patients): two patients died suddenly after diagnosis from aortic rupture and cardiac tamponade while waiting for surgery as detailed above; two others, who were surgically treated, died following multi-organ failure (one patient underwent urgent FET and one underwent urgent AR); the remaining patient died due to complications of a redo urgent ascending aorta replacement performed to treat retrograde AD extension after a hybrid repair (Table 4).

**Figure 2:**
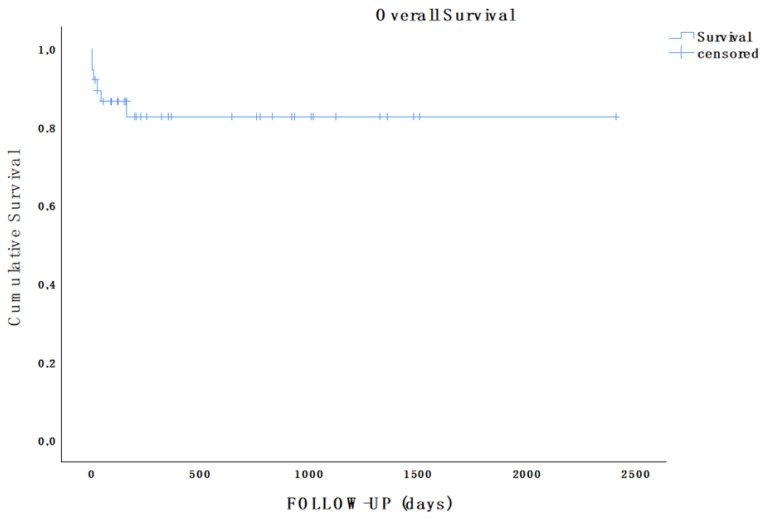
Kaplan–Meier estimate showing overall survival of the population in analysis during follow-up (mean follow-up, 16.9 months; range, 2 days–80.2 months).

During a mean follow-up of 16.9 months (range 2 days–80.2 months), the survival rate was 84.1% (32/38), as an additional patient who was surgically treated (elective FET and TEVAR) died from complications of chronic obstructive pulmonary disease 5 months after acute AD onset (Figure 2).

Aortic-related reinterventions were needed during follow-up in seven patients; see the details of these below:

a. In interventionally treated patients, aortic-related reinterventions during follow-up included two emergent operations (one TEVAR performed 4 days after FET for a DTA rupture, and one ascending aorta repair for retrograde extension of the dissection 8 days after a hybrid repair, as detailed above); three planned TEVAR procedures were completed for residual dissection with large communication within the lumens (one month after FET in two cases; three months after FET in one case).
b. In the medically treated group, two patients needed an aortic procedure during the follow-up: one of them underwent open thoracoabdominal aneurysm repair two years after the initial diagnosis; the other needed an axillo–femoral bypass to treat claudication following FL thrombosis and true lumen stenosis one month after initial diagnosis.

Preoperative anatomical data were analysed, comparing the two groups of patients (those who underwent surgery versus those underwent conservative management) to identify the independent predictors of intervention and mortality. The mean aortic diameter at the initial presentation was significantly higher in both zone 1 (37.6 ± 3.5 versus 34 ± 4.9, *p* = 0.043) and zone 2 (37.1 ± 4.4 versus 32.9 ± 6, *p* = 0.044) among patients needing intervention following medical treatment versus those responding to medical treatment alone (Table 5).

An increased in-hospital mortality rate (0% versus 30.8%, *p* = 0.023) was noted among patients who developed an indication for surgery after medical treatment (13/31, 41.9%).

The univariable and multivariable analysis (Table 6) did not identify any factor associated with increased 30-day and long-term mortality; this lack of an identification could be attributed to the small number of patients included in the study.

## 4. Discussion

NANB ADs are uncommon (3–11% of all acute AD) [7] and little is known about their clinical specificities and natural history.

In this multicentre study, we reviewed the treatment practices for NANB AAD across four high-volume aortic centres in Europe.

The present study, despite having limited data, provides additional clinical and anatomical information that may help practitioners to better define this rare disease; moreover, this study explores factors associated with complicated evolution of the condition.

The currently available data show that young (median age 59 years versus 65 and 67, respectively, in type A or B dissection) [3] males are the subpopulation most frequently affected, as seen in the present cohort [6]. No specific aetiologies were noted in this or previous series, with Marfan disease and hypertension comorbidities being well-known as being associated with AD [13,14]. 

Anatomical presentation in NANB may significantly differ from a case to another, both in terms of location of the primary tear and the distal extension. For instance, some series more frequently reported a primary tear located distal to the left subclavian artery (60.7%) [15]; others detected similar frequencies of NANB originating in the arch or more distally [16]; herein, we reported that most cases originated in Ishimaru zones 1 and 2, which is potentially a bias generated from the small sample.

If the experience in literature can provide an accurate reflection of clinical presentations, NANB patients seem to more often display a complicated initial presentation (malperfusion, pleural/cardiac effusion, retrograde dissection extending to the ascending aorta, impending rupture, and cardiogenic shock) [17]. From a clinical point of view, 10% of patients in the present series displayed signs of malperfusion at acute onset; however, a complicated initial presentation is reported to occur in up to a third of cases (29%), with an additional 6% of patients immediately presenting with signs of impeding rupture [7]. 

Currently, no consensus exists on the optimal treatment and timing for interventions. In our experience, admission to intensive care units for monitoring of blood pressure, analgesia, and antihypertensive therapy is indicated in all cases; these measures have been reported as the standard medical treatment in previous experiences. Interventional approaches are often required; frequently, operations are performed within two weeks of acute onset, as seen in [7,18] and as depicted above. Indeed, more than half of the patients in our series were scheduled for interventions, and most of these patients underwent surgery within two weeks of acute onset, raising concerns that earlier interventions might be indicated in this subset of dissection.

Commonly, people with complicated diseases undergo operations on an emergent/urgent basis; meanwhile, people with clinically stable AD normally undergo medical management. Triggers for intervention in the cases reported above (including hemodynamic instability, persistent pain in the setting of low blood pressure, risks of impending aortic rupture, secondary retrograde extension to the ascending aorta/root or end-organ ischemia) did not differ from those shown in clinical and radiological data as leading to operations in type A or type B dissections. Moreover, our data show that patients needing surgery after medical treatment had decreased survival at 30 days, meaning that there may be a subgroup of patients who may benefit from more aggressive treatments upon acute onset. For these reasons, we aimed to identify the factors associated with the worst-case clinical evolution, which could allow us to customize treatments and follow-ups for higher-risk patients. 

Hence, we investigated the factors associated with the need for intervention in initially uncomplicated diseases, revealing that an enlarged aortic diameter in zones 1 and 2 were associated with a need for intervention: those patients are more prone to complications.

In this cohort, patients needing surgery after medical treatment, at the baseline scan, presented with significantly enlarged aortic diameters in comparison with those who underwent medical treatment only. Since the population in study was too small to define a sensitive and specific cut-off, we could not identify a diameter threshold that could be predictive for the need for surgery; however, after both type A and type B aortic dissections, several independent risk factors for late intervention were already identified: maximum aortic diameter >40 mm; false lumen during the first two weeks of onset; persistence of the main entry tear, especially if it is located at the level of the aortic arch; the coexistence of Marfan disease; largely extensive dissection [18,19]. 

In our cohort, operations were performed regardless of the site of the primary tear. Accordingly, in most currently available studies, anatomic or goal-directed treatments are not reported, with only rare exceptions [9,15,17,19,20,21,22]. 

Rylinsky et al. [15] described results achieved from 30 endovascularly treated patients versus 4 surgically managed patients; they concluded that a tear-oriented operation should be performed from the outset to improve clinical outcomes and mortality rate.

A variety of strategies were adopted in the present cohort; however, none of the included patients underwent all-endovascular approaches which, conversely, represent the predominant strategies in the literature. This difference may help prove our belief that there is a bias in the present cohort. Indeed, Nauta et al. [22], in their retrospective review of the IRAD registry, analysed the results of patients (22 who were endovascularly treated versus 8 who underwent open surgery) who underwent type B dissection with an entry tear in the DTA and retrograde extension into the arch. They did not observe that retrograde involvement of the arch was an independent predictor of death; additionally, they praised the handiness and adaptability of the TEVAR procedure, even in cases of borderline-landing zone quality. They concluded that NANB AD approaches are superimposable to type B cases, as comparable outcomes can be achieved following similar treatments. However, the small number of patients in this cohort makes these conclusions tenuous.

In the largest series published so far on NANB, which compared open, endovascular, or non-interventional strategies, Liu j and colleagues [9] described the results achieved on 215 NANB patients (127 underwent endovascular treatment and 42 open surgery), reporting a higher thirty-day mortality rate with FET (7.1%) versus endovascular (1.6%) or medical (2.2%) strategies. During follow-up (median follow-up 39.1 months range 27.0–50.7), no additional deaths were recorded in the surgically treated patients, while a rate of 7.3% was recorded for those treated endovascularly, and a rate of 31.8% was recorded in the medically treated group (*p* < 0.01). Therefore, the authors concluded that endovascular approaches can be used to ensure optimal results only if a proper landing zone is achieved; they also described the FET technique as a valuable option in patients who are fit enough for surgery.

We reported an increased mortality in patients requiring surgery following best medical treatments, speculating that those patients may benefit from aggressive treatments from the beginning to lower their risks of complication; accordingly, a recent meta-analysis reported an increased 30-day mortality rate in medically treated patients when compared to those who underwent interventions (14% versus 3.6%) [7]. 

This cohort was too small to draw any definitive conclusions; effort should be made to better understand this rare condition and its clinical implications.

In this setting, we suggest that a largescale prospective study is needed to provide more substantial evidence for the standardized application of surgical/medical approaches according to the initial clinical and anatomical characteristics of the dissection.

## 5. Conclusions

This study provides descriptions of clinical aspects and current practices in the treatment of NANB cases in Europe. Most of the patients in this cohort had indications for surgery within two weeks from acute onset, demonstrating a frequently complicated course and raising questions surrounding the most appropriate timing for interventional management approaches. Although a diameter threshold was not identified, the baseline enlarged aortic diameters in zones 1 and 2 seemed to be associated with need for early interventions. Further study is needed to fully refine the indications for treatment in NANB patients, to study the independent risk factors for increased mortality risk and complications, and to identify a subgroup of patients that may benefit from more aggressive treatments from acute onset.

## 6. Limitations

This study has several limitations. To start with, the small patient cohort limits the statistical power of the findings, precluding robust significance testing and the stratification of major adverse events.

Additionally, the absence of a direct comparison group and the relatively short follow-up period further constrain the generalizability of our findings.

The inherent variability across the different included centres may have introduced confounding factors and potentially affected the reliability of the study results.

While these constraints limit generalizability, we restricted the number of predictors to clinically relevant variables to minimize the risk of overfitting; the analyses were conducted rigorously.

The findings remain valid within the scope of the data, providing meaningful insights that can inform future research; however, studies with larger sample sizes and longer follow-up periods are needed to confirm our results.

## Figures and Tables

**Table 1:** Demographics, comorbidities, and clinical presentations in NANB patients included in the analysis.

Baselines Data	Total = 38 n%
**Sex**	
Male	26 (68.4)
Female	12 (31.6)
**Age, years (mean, SD)**	60.5 (SD 12.9)
**Ethnicity**	
Caucasian	29 (76.3)
Asian	4 (10.5)
Black	5 (13.2)
**Cardiovascular risk factors**	
Hypertension	35 (92.1)
Active Smoker	8 (21.1)
Former smoker *	3 (7.9)
Diabetes Mellitus	2 (5.3)
Dyslipidaemia	11 (28.9)
**Comorbidities**	
Prior Stroke/TIA ^†^	4 (10.5)
COPD ^‡^	2 (5.3)
CAD ^§^	2 (5.3)
CKD ^‖^	4 (10.5)
Arrythmia	2 (5.3)
Marfan Syndrome	1 (2.6)
Alcohol abuse	1 (2.6)
Drug use	2 (5.3)
**Clinical presentation (one or more)**	
Uncontrolled hypertension	24 (63.2)
Chest/back pain	32 (84.2)
Abdominal pain	2 (5.3)
Neurological symptoms	3 (7.9)
Shortness of breath	1 (2.6)
Syncope	2 (5.3)
Malperfusion	4 (10.5)

Data are reported as n (%) or mean ± standard deviation (SD); * smoking weaned < 3 years. ^†^ TIA: transient ischemic attack; ^‡^ COPD: chronic obstructive pulmonary disease; ^§^ CAD: coronary artery disease; ^‖^ CKD: chronic kidney failure (GFR < 30 mL/min/1.73 m^2^).

**Table 2:** Anatomical data from the analysis of the baseline computed tomography angiography (CTA) of the included patients.

Anatomical Data	Total = 38 n%
**Primary entry tear at presentation**	
Zone 1	7 (18.4)
Zone 2	17 (44.7)
Zone 3	13 (34.2)
Zone 4	1 (2.6)
**Involvement of collateral branches**	
Brachiocephalic artery	1 (2.6)
Left common carotid	3 (7.9)
Left subclavian artery	2 (5.3)
Coeliac trunk	6 (15.8)
Superior mesenteric artery	8 (21.1)
Right renal artery	6 (15.8)
Left renal artery	18 (47.4)
Inferior mesenteric artery	11 (28.9)
**Mean Aortic Diameter (mm) at initial presentation**	**(median, IRQ)**
Zone 0	37 (7)
Zone 1	36 (7)
Zone 2	34.5 (6)
Zone 3	35 (11)
Zone 4	35 (11)
Zone 5	30.5 (7)
Zone 6	27 (8)
Zone 7	24.5 (6)

Data are reported as n (%) or median (IQR).

**Table 3:** Management strategies, indications, and timings for surgical treatments.

Management Strategy	Total = 38 (100) N (%)
**Treatment during the first 24 h**	
BMT *	31 (81.6)
Surgery	7 (18.4)
**Indication for surgical treatment during the first 24 h** **(one or more)**	Total = 13
Risks of impending aortic rupture/retrograde extension to the aortic root	6 (17.8)
Hemodynamic instability/persistent pain in the setting of low blood pressure	6 (17.8)
Clinical/laboratory signs of end organ malperfusion	1 (2.6)
Timing to Surgery after BMT * in days (mean, range)	13 (0–30)
Indication for surgical treatment after BMT *(one or more)	
Recurrent symptoms	2 (5.3)
Unfavourable anatomic evolution at the serial CTA ^†^ (rupture, proximal/distal extension of the dissection, aortic growth)	12 (38.7)
Clinical/laboratory signs of visceral/lower limb malperfusion	2 (5.3)

Data are presented as n (%); * BMT, best medical treatment; ^†^ CTA, computed tomography angiography.

**Table 4:** Clinical outcomes, mortality rates, and reintervention rates during in-hospital stays and during follow-up among the studied patients.

In-Hospital and Follow-Up Clinical Outcomes	
	Total = 38 (100%) N (%)
**30-day mortality**	5 (13.2)
**Medically treated complications**	
Stroke/TIA *	3 (7.9)
Myocardial infarction	-
Spinal cord ischemia ^†^	1 (2.6)
Pulmonary infection	11 (28.9)
Bowel ischaemia ^‡^	-
Renal failure ^§^	5 (13.5)
**Complications requiring surgical treatment**	8 (21.1)
**Survival rate during follow-up**	32 (84.1)
**Aortic-related reintervention**	7 (19.4)
**Length of stay (median, IQR)**	
ICU ^‖^ stay	9 (20)
Hospital stay	18 (25)
**Follow-up in days/months (mean, range)**	16.9 (2–80.2)

Data are presented as n (%) or mean ± standard deviation (SD) or median (interquartile range [IQR]); * TIA, transient ischemic attack; ^†^ complete paralysis of the legs, total loss of pinprick and vibratory sensation caudal to the T9 level, and laxity of sphincters; ^‡^ mesenteric ischemia or ischemic colitis with or without bowel resection; ^§^ requiring permanent or definitive dialysis; ^‖^ ICU, intensive care unit.

**Table 5:** Outcomes achieved in medically treated patients versus those who underwent surgery after BMT; variables associated with need for surgery after first-line BMT.

Outcomes Comparison Between Interventionally and Conservatively Treated Groups			
Outcomes	Need for Surgery After BMT		
	NO	YES	***p***
**30-day mortality**	-	4 (30.8)	**0.023**
**Complication**			
Stroke/TIA *	1 (5.6)	2 (15.4)	0.558
Myocardial infarction	-	-	-
Spinal cord ischemia ^†^	-	-	-
Pulmonary infection	-	6 (46.2)	0.002
Bowel ischaemia ^‡^	-	-	-
Renal failure ^§^	1 (5.6)	3 (25.0)	0.274
**Aortic-related intervention during follow-up**	3 (16.7)	2 (18.2)	0.917
**Thrombosis FL**	9 (52.9)	9 (81.8)	0.226
**Death during follow-up**	-	1 (8.3)	0.400
**Length of stay (mean, range)**			
ICU ^‖^	8 (1–30)	29 (2–59)	**0.010**
Hospital Stay	13 (6–45)	38 (2–54)	**0.012**
**Follow-up (mean, range)**	630.4 (18–2406)	445.5 (2–1359)	0.415
	**Need for surgery after BMT**		
	NO (total = 18)	YES (total = 13)	***p***
**Female**	6 (33.3)	5 (38.5)	0.768
**Age (mean, SD)**	57.6 (11.4)	66.1 (12.5)	0.059
**Ethnicity**			
Caucasian	13 (72.2)	11 (84.6)	
Asian	2 (11.1)	1 (7.7)	
Black	3 (16.7)	1 (7.7)	
**Hypertension**	15 (83.3)	13 (100)	0.245
**Dyslipidaemia**	5 (27.8)	5 (38.5)	0.530
**COPD**	1 (5.6)	1 (7.7)	0.811
Prior stroke/TIA *	2 (11.1)	2 (15.4)	0.726
**Diabetes mellitus type II**	-	1 (7.7)	0.419
**CAD**	2 (11.1)	-	0.497
**Chronic kidney failure (GFR < 30 mL/min/1.73 m^2^)**	1 (5.6)	2 (15.4)	0.558
**Atrial fibrillation**	-	1 (7.7)	0.419
**Aortic diameter (median, IQR)**			
Zone 0	36.5 (9)	37 (5)	0.146
Zone 1	34 (7)	37 (3)	**0.043**
Zone 2	32.5 (7)	36 (6)	**0.044**
Zone 3	35 (8)	37 (10)	0.472
Zone 4	34.5 (12)	35 (15)	0.366
Zone 5	30 (10)	31 (5)	0.448
Zone 6	24.5 (7)	28 (8)	0.126
Zone 7	22.5 (8)	25 (4)	0.431

Data are presented as n (%) or mean ± standard deviation (SD); * TIA, transient ischemic attack; ^†^ complete paralysis of the legs, total loss of pinprick and vibratory sensation caudal to the T9 level, laxity of sphincters; ^‡^ mesenteric ischemia or ischemic colitis with or without bowel resection; ^§^ requiring permanent or definitive dialysis; ^‖^ ICU, intensive care unit. *p* values < 0.05 are reported in bold.

**Table 6:** Univariable and multivariable analysis on the factors associated with increased risk of 30-day and long-term mortality.

Factors Associated with Increased Risk of Mortality				
	**Univariable analysis**		**Multivariable analysis**	
	**OR (95% CI)**	***p***	**OR (95% CI)**	***p***
Male	4.000 (0.571–28.011)	0.301	7.658 (0.291–201.431)	0.222
Hypertension	1.167 (1.019–1.336)	1.000		
Smoking	-	1.000		
Diabetes	-	1.000		
Dyslipidaemia	1.778 (0.254–12.449)	0.559	4.445 (0.115–171.213)	0.423
Stroke/TIA	2.500 (0.207–30.215)	0.459		
COPD	-	1.000		
CAD	-	1.000		
Renal failure	-	1.000		
Arrythmia	-	1.000		
Marfan Syndrome				
Alcohol abuse	**-**	1.000		
Drug use	-	1.000		
Previous aortic surgery	-	1.000		
Entry tear				
Zone 1	-	1.000		
Zone 2	6.154 (0.617–61.371)	0.152		
Zone 3	0.438 (0.044–4.378)	0.643		
Zone 4	-	1.000		
Proximal extent of the AD	0.339 (0.034–3.376)	0.630		

TIA, transient ischemic attack; COPD, chronic obstructive pulmonary disease; CAD, coronary artery disease; AD, aortic dissection; *p* values < 0.05 were considered to be significant.

## Data Availability

Data underlying this article will be shared on reasonable request to the corresponding author.
